# Variation in Fitness of the Longhorned Beetle, *Dectes texanus*, as a Function of Host Plant

**DOI:** 10.1673/031.010.20601

**Published:** 2010-12-08

**Authors:** J.P. Michaud, Angela K. Grant

**Affiliations:** Department of Entomology, Kansas State University, Agricultural Research Center, Hays, KS, 67601, 785-6253425

**Keywords:** body size, *Glycines max*, *Helianthus annuus*, host plant, longevity, oviposition, survival

## Abstract

*Dectes texanus* LeConte (Coleoptera: Cerambycidae) has become a serious pest of two different crops in the American Midwest, sunflower, *Helianthus annuus* L. and soybean, *Glycines max* (L.). Laboratory and field studies were used to compare the effects of these two host plants on *D. texanus* life history and behavior. Insects from soybean were 40–60% smaller than those from sunflower and larval weight at collection was strongly correlated with survival to adulthood, whereas it was not in sunflower, suggesting that body size was more limiting to immature survival in soybean. Pupal weights increased more rapidly with increasing stem diameter in soybean than in sunflower and the correlation was stronger, indicating that body size was more limited by plant size in soybean. Adults collected as larvae from soybean had shorter longevities when starved, fed soybean, or fed an alternating diet of soybean and cultivated sunflower, than did those collected from sunflower, suggesting a negative larval legacy of soybean on adult fitness. Adult beetles that developed in soybean lived longer when fed soybean than when starved, but an adult diet of sunflower doubled longevity compared to soybean for beetles that developed in sunflower, and tripled it for those that developed in soybean. An adult diet of wild *H. annuus* yielded survivorship equivalent to cultivated *H. annuus* in one trial, and slightly lower in another. Larval host plant did not influence the numbers of ovipunctures or eggs laid by females in field trials, but adult diet did. Sunflower-fed females punctured more, and laid more eggs, on sunflowers than on soybeans in field cages and the reverse trend was evident, but not significant, in soybean-fed females. It can be concluded that *H. annuus* is a superior food source to *G. max* for both larval and adult *D. texanus*, and that wild sunflowers may represent a valuable food for adults during the pre-reproductive period, prior to invasion of soybean fields, even though they rarely host larvae. We also showed that stable isotope ratios of N can be used to distinguish the larval host plant of beetles regardless of their adult diet.

## Introduction

The long-horned beetle *Dectes texanus* LeConte (Coleoptera: Cerambycidae) is a serious pest of cultivated sunflowers and soybeans throughout much of its range in the United States (Hatchett et al. 1975, Rogers 1985, Michaud and Grant 2005). Although there appears to be little direct impact of larval boring on plant productivity in soybean (Richardson 1975; Andrews and Williams 1988) and no impact in sunflowers (Michaud et al. 2007a), yield losses may occur as a result of pre-harvest lodging. Lodging is often a problem in sunflowers but can be greatly exacerbated as a consequence of *D. texanus* larvae girdling stalks at their base in preparation for overwintering. Since larvae are solitary and aggressive toward conspecifics, girdling behavior is thought to represent a defensive tactic for securing an overwintering site at the base of the plant (Michaud and Grant 2005). Whenever a large proportion of plants are infested and harvest is delayed, yield losses of both crops can be substantial.

Previous work has indicated that soybean represents an inferior larval host plant relative to cultivated sunflower. Not only does development in soybean result in much smaller beetles, but the plant is generally less preferred for feeding and oviposition by adult females (Michaud and Grant 2005). Cultivated sunflowers can effectively be used as a trap crop to reduce infestation in soybeans (Michaud et al. 2007b). In previous work (Michaud and Grant 2005), host plant acceptance by females collected as larvae from sunflower stubble, and subsequently fed on either soybean or sunflower petioles, was tested by caging individuals on each of two plants in sequence in the field. It was found that feeding behavior in the field was correlated with oviposition behavior and that females fed soybean in the laboratory fed and ovipunctured more on soybean in field trials than females fed sunflower during the preoviposition period. However, there was an effect of the order in which plants were presented to females and this complicated the interpretation of results. Furthermore, we did not test females collected as larvae from soybean stubble, nor were the fitness consequences of soybean versus sunflower as an adult food plant examined. The present study was designed to resolve some of these issues.

In this study, the effect of adult food plant on longevity was examined and the oviposition responses of a larger number of adult females was field-tested, this time using individuals obtained as larvae from both sunflower and soybean, and then fed either the same or the alternate host plant as adults. Furthermore, the effect of body size on larval survival was examined in more detail and the relationship between stalk diameter and pupal weight was analyzed using data from a number of cohorts of *D. texanus* obtained from both host plants over a series of years. In addition, stable isotope ratios of nitrogen were measured and tested as a means of distinguishing the larval host plant of adult beetles.

## Materials and Methods

### Body size and larval survival

Overwintered larvae of *D. texanus* were collected from field stubble of soybean and sunflower in Ellis and Finney Counties, Kansas during late winter or early spring over a period of four years (2005–2008). The dates reported for cohorts refer to the year of collection and emergence as adults, rather than the year of their development within plants. Sample sizes for the eight cohorts collected over the four years, respectively, were as follows: sunflower —— 111, 89, 61 and 140; soybean —— 149, 93, 83 and 139. Stalks were split and the larvae removed and weighed before isolating them individually in plastic Petri dishes (5.5 cm diameter) containing a piece of filter paper. Dishes containing larvae were individually numbered and placed on trays in a climate-controlled growth chamber set to 24°° C and 16:8 L:D day length under Philips ‘‘coolwhite’’ fluorescent lighting, unless otherwise specified. Relative humidity ranged from 50– 60%. Larvae were examined daily and pupal weights recorded within 24 h of pupation. Sex was determined on the day of pupation according to the characters described by Hatchett et al. (1975).

### Body weight versus stem diameter

Six cohorts of larvae (three each from sunflower and soybean), corresponding to those obtained in 2006, 2007 and 2008, were used to examine the relationship between stalk diameter of the larval host plant and insect weights. The stalk diameter of each plant was estimated at the soil line by taking maximum and minimum measurements with a digital caliper and averaging the two values. The stalks were split to extract the larvae and these were immediately weighed and isolated in Petri dishes that were then held under climate controlled conditions (as above). Pupal weights were used for correlation with stalk diameters since they were standardized with respect to stage of development. As larvae undergo a variable number of molts following collection, their stage of development is indeterminate prior to pupation.

### Adult diet

The effect of adult diet on longevity was examined in paired cohorts of *D. texanus*, one obtained from sunflower and the other from soybean, in 2005, 2006 and 2008. Seeds of soybean (Asgrow 3003) and sunflower (Triumph 665) were germinated in metal trays (8 ×× 26 ×× 36 cm) filled with potting soil and held in a greenhouse under natural light conditions to produce seedlings for use as adult food. Petioles of wild *H. annuus* were harvested as required from plants in a nearby pasture. In 2005 and 2008, male and female adults of each cohort were divided randomly upon emergence into three diet treatments: (1) soybean petioles, (2) cultivated sunflower petioles and (3) wild sunflower petioles. In 2006, male and female adults of each cohort were divided randomly upon emergence into four diet treatments: (1) starvation, (2) soybean petioles, (3) cultivated sunflower petioles and (4) soybean and cultivated sunflower petioles provided alternately on each feeding day to encourage consumption of both plants. All adult beetles were checked daily for mortality and, with the exception of starvation treatments, received fresh plant material every second day until they died.

Samples of insects from the 2006 experiment were dried and weighed and subjected to analysis of nitrogen isotope content. Samples were analyzed in the laboratory of R. Lee (School of Biological Sciences, Washington State University). Dried sample was added to tin capsules and combusted in a Eurovector (www.eurovector.it) elemental analyzer. The resulting N_2_ and CO_2_ gases were separated by gas chromatography and admitted into the inlet of a GV instruments Isoprime isotope ratio mass spectrometer (IRMS) (www.thermofisher.com) for determination of ^15^N/^14^N and ^13^C/^12^C ratios. Typical precision of analyses was ±± 0.5 ‰‰ for δ?^15^N and ±± 0.2 ‰‰ for δ? ^13^C where δ? = 1000 ×× (Rsample/Rstandard) -1 ‰‰, where R = ^15^N /^14^N, the standard for δ? ^15^N is atmospheric nitrogen and Peedee belemnite (PCB) for δ? ^13^C. Delta values correlate with ^15^N and ^13^C content of samples. Higher δ? values correspond with higher ^15^N and ^13^C contents. Egg albumin was used as a daily reference material. Data were subjected to a two-way ANOVA with larval host plant and adult diet as independent variables.

### Oviposition

The effects of larval host plant and adult diet on the oviposition behavior of female beetles was tested in the field in the summers of 2005 and 2006. Overwintered larvae were obtained from sunflower and soybean stubble and held until adult emergence (as described above). Upon emergence, female adults of each larval host plant group were arbitrarily assigned to one of two adult feeding treatments; stems of either cultivated sunflower or soybean seedlings grown in a greenhouse (as described above). All males were fed sunflower stems. When beetles were 8–12 days old, males and females were brought together in pairs in Petri dishes (9.5 cm diameter) and allowed to copulate for a period of 2 – 5 h. Females were then isolated from males and used in field trials when they were 18–24 days old.

Field-grown sunflower (Triumph 665) and soybean (Asgrow 3003) plants in similar vegetative growth stages were selected for oviposition trials and carefully examined to ensure they had no pre-existing oviposition punctures by wild *D. texanus* in any of their petioles. Cylindrical wire-frame, fabric mesh cages (30 cm diameter) were used to confine female beetles on plants (www.lucina.freeserve.co.uk/index.html). The height of the cage was adjustable from 30 -100 cm, according to the height of the plant, by altering the numbers of slotted frame members. The lower edges of the fabric were buried in the soil and a large, zippered opening permitted access for introduction and removal of beetles. Between 3 July and 27 July, individual mated females were introduced to cages in early morning hours and each remained on a plant for a period of 48 h. Following recovery and removal of the female, the plant was uprooted and taken to the laboratory. The stem and petioles of each plant were carefully examined for oviposition punctures and each puncture was sliced open with a surgical scalpel to determine the presence or absence of an egg.

## Results

### Body size and host plant

**Figure 1.**
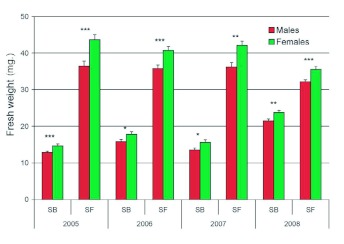
Mean (+SEM) adult fresh weights upon emergence of male and female Dectes *texanus* collected as larvae from stubble of either soybean (SB) or cultivated sunflower (SF) in each of four years. Among years, sunflower individuals of both sexes were lighter in 2008 than in other years and the order of weights for soybean individuals of both sexes was 2008 > 2006 > 2007 = 2005. Asterisks indicate cohorts in which females were significantly heavier than males (**, α? ≤? 0.05; ****, α? ≤? 0.01; ******, α? ≤? 0.001). In all years, beetles collected from sunflower were heavier than those collected from soybean, regardless of sex (LSD, α? < 0.001 in all cases). High quality figures are available online.

The mean fresh weights of adults at emergence are reported for all eight cohorts in Figure 1. The 3-way ANOVA of adult fresh weight with ‘‘year’’, ‘‘host plant’’ and ‘‘sex’’ as independent variables was significant (*F*_(15,811)_ = 181.21, *P* < 0.001). Effects of year (*F*_(3,811)_ = 2.65, *P* = 0.048), host plant (*F*_(1,811)_ = 2230.04, *P* < 0.001) and sex (*F*_(1,811)_ = 70.76, *P* < 0.001) were all significant, as were the interactions year**host plant (*F*_(3,811)_= 74.02, *P* < 0.001) and host plant**sex (*F*_(1,811)_ = 14.17, *P* < 0.001). The interactions year**sex (*F*_(3,811)_ = 0.84, *P* = 0.472) and year**host plant**sex (*F*_(3,811)_ = 1.36, *P* = 0.253) were not significant. There were significant differences among years for the weight of males (*F*_(3,811)_ = 4.36, *P* = 0.005) and females (*F*_(3,192)_ = 11.88, *P* < 0.001) from sunflower, individuals of both sexes being significantly lighter (LSD, α? = 0.05) in 2008 than in other years. There were also significant differences among years for the weight of males (*F*_(3,217)_ = 70.00, *P* < 0.001) and females (*F*_(3,198)_ = 47.32, *P* < 0.001) from soybean. The weight of both sexes was 2008 > 2006 > 2007 = 2005.

In 2005, female beetles were heavier than males whether collected as larvae from sunflower (*F*_(1,109)_= 13.88, *P* < 0.001) or from soybean (*F*_(1,146)_ = 11.53, *P* = 0.001) and adults of both sexes were significantly heavier when obtained from sunflower (males: *F*_(1,87)_ = 432.65, *P* < 0.001; females: *F*_(1,91)_ = 414.09, *P* < 0.001). In 2006, female beetles were again heavier than males whether collected as larvae from sunflower (*F*_(1,87)_ = 12.90, *P* = 0.001) or from soybean (*F*_(1,91)_ = 4.22, *P* = 0.043) and adults of both sexes were significantly heavier when obtained from sunflower (males: *F*_(1,87)_ = 300.07, *P* < 0.001; females: *F*_(1,91)_ = 335.50, *P* < 0.001).

In 2007, female beetles were heavier than males among insects collected from sunflower (*F*_(1,55)_ = 10.99, *P* = 0.002) but the difference was only marginally significant for insects collected from soybean (*F*_(1,61)_ = 3.93, *P* = 0.052) and adults of both sexes were significantly heavier when obtained from sunflower (males: *F*_(1,50)_ = 244.12, *P* < 0.001; females: *F*_(1,66)_ = 359.88, *P* < 0.001). In 2008, female beetles were heavier than males when collected from sunflower (*F*_(1,129)_ = 12.92, *P* < 0.001) and from soybean (*F*_(1,117)_ = 7.91, *P* = 0.006). Sunflower insects were once again heavier than soybean insects regardless of sex (males: *F*_(1,123)_ = 183.16, *P* < 0.001; females: *F*_(1,123)_ = 146.86, *P* < 0.001).

### Larval weight and survival

**Figure 2.**
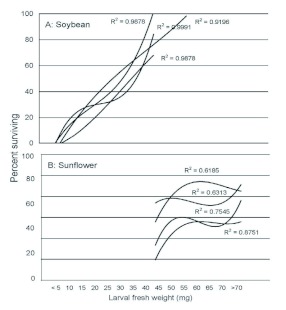
Cubic regressions of larval fresh weights versus percentage of various size classes surviving to adult for eight cohorts of *Dectes texanus*, four each from soybean (A) and cultivated sunflower (B). Regressions were significant for soybean cohorts (α? < 0.005 in all cases), but not for sunflower cohorts (α? > 0.05 in all cases). High quality figures are available online.

The survival of larvae (to emergence as adult) in different weight classes was analyzed as a frequency distribution in 5 mg intervals for the various cohorts collected as larvae from soybean and sunflower. Cubic regressions yielded the highest r^2^ values for soybean cohorts (2005: *F* = 80.75, *P* = 0.002; 2006: *F* = 1151.52, *P* < 0.001; 2007: *F* = 80.66, *P* = 0.002; 2008: *F* = 19.06, *P* = 0.004; Fig 2A). Cubic regressions also provided higher r^2^ values for sunflower cohorts than other models tested. Although these regressions were not significant (2005: *F* = 7.01, *P* = 0.072; 2006: *F* = 1.71, *P* = 0.335; 2007: *F* = 3.07, *P* = 0.191; 2008: *F* = 1.08, *P* = 0.513), we plotted them for sake of comparison to soybean cohorts (Figure 2B).

### Pupal weight versus stem diameter

**Figure 3.**
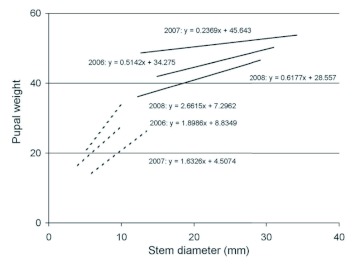
Linear regressions of pupal fresh weights against larval host plant stem diameters for six cohorts of *Dectes texanus*, three each from cultivated sunflower (solid lines) and soybean (dashed lines). Regressions were significant for soybean cohorts (α? < 0.001 in all cases) and two of the three sunflower cohorts (2006: α? < 0.05, 2007: ns, 2008, α? < 0.001). High quality figures are available online.

Linear regression revealed positive correlations between host plant stem diameter and *D. texanus* pupal weight in all cohorts, and all but one were significant (2006 sunflower: *F*_(1,87)_ = 4.44, *P* = 0.038, r^2^ = 0.049; 2006 soybean: *F*_(1,84)_ = 14.98, *P* < 0.001, r^2^ = 0.151; 2007 sunflower: *F*_(1,59)_ = 1.21, *P* = 0.275, r^2^ = 0.020; 2007 soybean: *F*_(1,81)_ = 32.74, *P* < 0.001, r^2^ = 0.288; 2008 sunflower: *F*_(1,142)_ = 16.71, *P* < 0.001, r^2^ = 0.105; 2008 soybean: *F*_(1,137)_ = 32.76, *P* < 0.001, r^2^ = 0.193; Figure 3). All cohorts exhibited substantial variation in body size for a given stem size, such that a relatively small proportion of variation was explained. However, year by year comparisons of cohorts from sunflower and soybean all failed tests for homogeneity of regression (α? = 0.01 in all cases, Sheskin 2000) indicating that the regression lines for cohorts collected from soybean had significantly steeper slopes than those from sunflower in all three years.

### Adult diet and longevity

In 2005, there was a significant interaction between larval host plant and adult diet with respect to adult longevity (*F*_(2,223)_ = 15.39, *P* < 0.001) but interactions between larval host plant and sex (*F*_(1,223)_ = 0.03, *P* = 0.872), adult diet and sex (*F*_(2,223)_ = 1.36, *P* = 0.259) and the three way interaction (*F*_(2,223)_ = 1.68, *P* = 0.188) were not significant.

**Figure 4.**
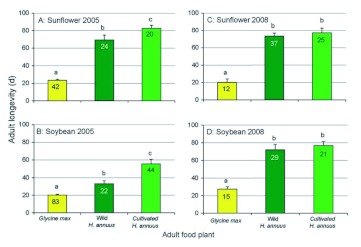
Longevities of *Dectes texanus* adults obtained as larvae in 2005 (A and B) and 2008 (C and D) from either cultivated *Helianthus annuus* (A and C) or *Glycine max* (B and D) and fed an adult diet of *G. max*, wild *H. annuus*, or cultivated *H. annuus*. Columns bearing the same letter were not significantly different among treatments within a cohort (LSD, α? > 0.05). Numbers indicate cohort sample sizes. Insects obtained as larvae from cultivated *H. annuus* lived longer than those obtained from *G. max* on all three adult diets in 2005 (α? < 0.05 in all cases), but there was no effect of larval host plant in 2008 (α? > 0.05 in all cases). High quality figures are available online.

Sex did not affect adult longevity in 2005 whether insects were derived from sunflower (*F*_(1,84_) = 2.61, *P* = 0.109) or from soybean (*F*_(1,147)_ = 0.12, *P* = 0.726). There was a significant effect of adult diet on the longevity of beetles collected as larvae from sunflower (*F*_(2,83)_ = 109.35, *P* < 0.001, Figure 4a), the order of suitability being soybean < wild sunflower < cultivated sunflower. There was also a significant effect of adult diet on the longevity of beetles collected from soybean (*F*_(2,146)_ = 49.82, *P* < 0.001, Figure 4b), the order of suitability being the same. Beetles collected as larvae from sunflower lived longer than those collected from soybean whether they were fed soybean (*F*_(1,123)_ = 5.485, *P* = 0.021), wild sunflower (*F*_(1,44)_ = 30.14, *P* < 0.001), or cultivated sunflower (*F*_(1,62)_ = 13.09, *P* = 0.001).

In 2008, there were no significant interactions between sex and adult diet (*F*_(2,138)_ = 1.65, *P* = 0.196), sex and larval host plant (*F*_(1,138)_ = 0.07, *P* = 0.795), or adult diet and larval host plant (*F*_(2,138)_ = 0.33, *P* = 0.721) with respect to their effects on longevity and the three-way interaction was likewise not significant (*F*_(2,138)_ = 1.34, *P* = 0.265).

Neither sex (*F*_(1,162)_ = 3.07, *P* = 0.081) nor larval host plant (*F*_(1,162)_ = 0.11, *P* = 0.740) had a significant effect on longevity in 2008, but data were plotted separately by larval host to conform to 2005 data (Figure 4c,d). The overall effect of adult diet was significant (*F*_(2,138)_ = 51.13, *P* < 0.001). Adults fed cultivated sunflower did not differ in longevity from those fed wild sunflower, but those fed soybean died earlier.

In 2006, there was a significant interaction between sex and adult diet with respect to adult longevity (*F*_(3,166)_ = 5.03, *P* = 0.002). The interaction between sex and larval host plant was not significant (*F*_(3,166)_ = 3.19, *P* = 0.076), nor was that between larval host plant and adult diet (*F*_(3,166)_ = 2.05, *P* = 0.109), but the three-way interaction (larval host plant**sex**adult diet) was significant (*F*_(3,166)_ = 5.38, *P* = 0.001).

**Figure 5.**
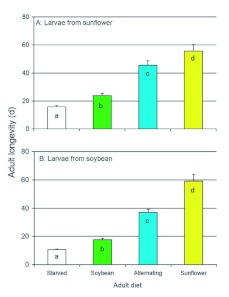
Longevities of *Dectes texanus* adults obtained as larvae from either cultivated sunflower (A) or soybean (B) and either starved, fed soybean, fed an alternating diet of cultivated sunflower and soybean, or fed cultivated sunflower. Columns bearing different letters were significantly different among treatments within a cohort (LSD, α? < 0.05). With the exception of the cultivated sunflower diet, insects obtained from sunflower lived longer than those obtained from soybean in all treatments (α? ≤? 0.002 in all cases). High quality figures are available online.

Sex did not affect the longevity of beetles in 2006 whether they were collected from sunflower (*F*_(1,87)_ = 0.12, *P* = 0.729) or from soybean (*F*_(1,91)_ = 0.46, *P* = 0.500). There was a significant effect of adult diet on the longevity of insects obtained as larvae from sunflower (*F*_(3,85)_ = 52.44, *P* < 0.001, Figure 5a) and the same was true for insects obtained from soybean (*F*_(3,89)_ = 84.32, *P* < 0.001, Figure 5b). Insects obtained as larvae from sunflower survived better than those from soybean when starved (*F*_(1,50)_ = 37.45, *P* < 0.001), fed soybean (*F*_(1,52)_ = 10.45, *P* = 0.002), or an alternating diet of soybean and sunflower (*F*_(1,37)_ = 4.98, *P* = 0.032), but did not differ when fed sunflower (*F*_(1,35)_ = 0.28, *P* = 0.602).

### Host plant, adult diet, and nitrogen isotope ratios

**Figure 6.**
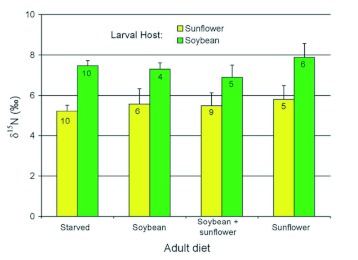
Mean (+SEM) ratios of stable nitrogen isotopes in adult *D. texanus* obtained from either cultivated sunflower or soybean and fed one of four different diets as adults. Numbers on bars correspond to numbers of insects sampled. There was no significant effect of adult diet, but δ? ^15^N was proportionally higher in beetles obtained as larvae from soybean than from sunflower (*P* < 0.001). High quality figures are available online.

Analysis of nitrogen isotopes revealed no effect of adult diet (*F*_(3,45)_ = 0.92, *P* = 0.674), but a significant effect of larval host plant (*F*_(3,45)_ = 22.47, *P* < 0.001, Figure 6). Pooling data across adult diets, δ? ^15^N was proportionally higher in beetles obtained from soybean than from sunflower (mean ±± SEM = 7.43 ±± 0.23 vs 5.46 ±± 0.27; *F*_(1,51)_= 30.64, *P* < 0.001).

### Adult diet and oviposition behavior

Only data from females that laid at least one egg in their field trial were included in this analysis. There was no effect of larval host plant on either the number of ovipunctures or eggs laid in sunflower plants (*F*_(1,77)_ = 0.12, *P* = 0.727 and *F*_(1,77)_ = 0.098, *P* = 0.755, respectively) or in soybean plants (*F*_(1,76)_ = 0.85, *P* = 0.362 and *F*_(1,76)_ = 0.90, *P* = 0.347, respectively) so larval host plant was ignored for analysis of effects of adult diet on oviposition behavior. The two-way ANOVA revealed a significant interaction between adult food plant and oviposition plant for both number of ovipunctures (*F*_(3,153)_ = 6.37, *P* = 0.013) and number of eggs laid (*F*_(3,153)_ = 6.43, *P* = 0.012) so these factors were analyzed separately.

**Figure 7.**
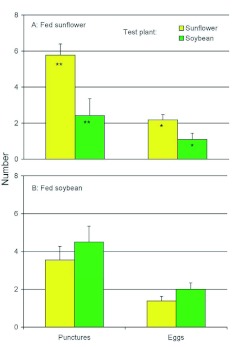
Numbers of ovipunctures made and eggs laid by *Dectes texanus* females fed an adult diet of either cultivated sunflower (A) or soybean (B) prior to being caged for 48 h on a test plant in the field. Asterisks indicate a significant effect of adult diet (**, α? ≤? 0.05; ****, α? ≤? 0.01). Sunflower-fed females punctured more, and laid more eggs in, sunflower plants than on soybean plants (α? < 0.05 in both cases), but responses of soybean-fed females were not significantly different between host plants (α? > 0.05 in both cases). High quality figures are available online.

Females fed sunflower as adults made more ovipunctures (*F*_(1,77)_ = 8.11, *P* = 0.006) and laid more eggs (*F*_(1,77)_ = 4.33, *P* = 0.0.041) on sunflower plants than on soybean (Figure 7a). However, females fed soybean as an adult diet made similar numbers of ovipunctures (*F*_(1,76)_ = 0.61, *P* = 0.438) and laid similar numbers of eggs (*F*_(1,76)_ = 2.12, *P* = 0.150) on each type of plant (Figure 7b). Sunflower-fed females made more ovipunctures (*F*_(1,110)_ = 5.47, *P* = 0.021) and laid more eggs (*F*_(1,110)_ = 4.57, *P* = 0.035) in sunflower plants than did soybean-fed females, but differences were not quite significant for either number of ovipunctures (*F*_(1,43)_ = 2.77, *P* = 0.103) or number of eggs laid (*F*_(1,43)_ = 3.46, *P* = 0.070) in soybean plants.

## Discussion

Larvae of *D. texanus* obtained from soybean averaged 40–60 % lighter in weight than those from cultivated sunflower (Figure 1), consistent with previous reports (Michaud and Grant 2005; Niide et al. 2006). Larval weight appeared to have a much stronger effect on survival in soybean than in sunflower as revealed by significant curvilinear regressions, higher r^2^ values, and steeper curve slopes (Figure 2). It may be concluded that exploitation of soybean restricts many *D. texanus* larvae to body sizes that often approach the lower size limit for survival, whereas larvae are sufficiently large in cultivated sunflower that survival probability is no longer a function of body size. The slopes of regression lines of stalk diameter on pupal weight were significantly steeper in soybean cohorts than in sunflower cohorts (Figure 3), indicating that plant size is more of a constraint on *D. texanus* body size in the former host plant. Similarly, the relatively larger size of soybean insects in 2006 and 2008 compared to 2005 and 2007 is likely attributable to generally larger plant size in the fields sampled. It is not clear why sunflower insects were smaller in 2008, but rainfall was unusually heavy during the 2007 growing season, possibly in excess of the optimum for sunflower plants.

The chemical composition of sunflower pith was analyzed by Yin et al. (2007) and found to be comprised of 47.4% cellulose, 9.4% hemicellulose, 6.0% pectin, 3.5% lignin and 1.0% soluble sugars, the remainder comprising water and ash. From these values, it is apparent that *D. texanus* larvae obtain protein for growth and development from pectin, the only source of nitrogen in sunflower pith. Comparable information is not available for soybean pith, but even though physical space and quantity of resources may be limiting for larvae in soybeans, it is possible that the pith of this leguminous plant is richer in nitrogen than that of composites such as sunflower. Stalk boring insects are often nitrogen-limited (e.g., Bergvinson et al. 1997; Sharma et al. 2007) and *D. texanus* may be able to complete development in soybean at smaller body sizes and with consumption of smaller volumes of food if this is the case. Indeed, larvae from soybean plants have a noticeably dark yellow coloration that may well arise from their ingestion of nitrogen-rich compounds.

The ratio of stable nitrogen isotopes in adult beetles was distinct for the two larval host plants (Figure 6) and likely reflects the incorporation of nitrogen fixed from the atmosphere by the root nodules of soybean, a process that does not occur in sunflower. The nitrogen signature of the larval host plant was not compromised by consumption of alternative plant material during the adult stage, likely because body mass is accumulated during larval feeding. Although some nitrogen turnover during adult life would be expected in soft body parts, deposits in dead tissues such as chitin presumably remain stable, making the insect cuticle the best choice for IRMS samples when the larval host plant is of interest.

The short life of adults on a diet of soybean petioles (Figure 4) confirms previous observations (Michaud and Grant 2005) that adults feed sparingly on soybean and barely live long enough to attain reproductive maturity when fed exclusively on this plant. The present data show that an adult diet of soybean provides only a slight benefit over complete starvation (Figure 5), although survival is substantially improved when the beetles are able to supplement their diet with superior food, as reflected in the alternating diet treatment. We conclude that adult beetles probably depend on alternative food plants during the maturation phase (2–3 weeks) prior to invading soybean fields. There was also a legacy of larval diet on adult performance; beetles developing on sunflower tended to live longer as adults than those that developed on soybean, especially when starved or provided low quality food (Figures 4 and 5). The exception was for 2008 in which longevity was not diminished by a larval diet of soybean in any treatment (Figure 4c and d). Larvae from soybean were notably larger in the 2008 negative larval legacy of soybean in the 2005 and 2006 experiments may be associated with larvae failing to reach a critical size as a result of resource limitation during development.

Wild *H. annuus* plants express considerable antixenosis to *D. texanus* females and rarely host larvae of this cerambycid in Kansas (Michaud and Grant 2005; Michaud and Grant 2009), although other species can be found. Petioles of wild *H. annuus* are tougher than their cultivated counterparts, less succulent, and exude more than four times the amount of resinous material (by weight) per cm diameter when severed, properties that may confer resistance to oviposition (Michaud and Grant 2009). Nevertheless, it is clear from the results presented here that wild sunflower can serve as a valuable food source for adults prior to their arrival in crop fields, particularly if that crop is soybean. In the 2005 trial, beetles from both larval host plants had shorter lives when fed petioles of wild *H. annuus* compared to cultivated, suggesting they may be somewhat less suitable, but this was not the case in the 2008 trial. The soybean cohort was comprised of especially large insects in 2008, whereas those in the sunflower cohort were especially small, so we suspect these inconsistent results reflect natural variation in the wild sunflowers used for food, rather than any factor specific to the insect cohorts.

Larval host plant had no effect on adult oviposition behavior, but feeding on a plant as an adult during the pre-reproductive period tended to increase its acceptability for oviposition in the field, although the effect was more pronounced for females fed sunflower (Figure 7). The lack of significant differences for soybean-fed females is consistent with previous work that indicated feeding on sunflower as an adult reduces the subsequent acceptability of soybean for oviposition, whereas soybean feeding does not reduce the subsequent acceptability of sunflower, although it may increase the acceptability of soybean (Michaud and Grant 2005; Michaud et al. 2007b). The higher activity of sunflower-fed females compared to soybean-fed females on the preferred host probably reflects the greater overall vigor of the former individuals.

In summary, our data indicate that the survival of immature *D. texanus* is correlated with body size in soybean cohorts, but not in sunflower cohorts that are substantially larger. The steeper slopes of regression lines and stronger correlations between body size and stem diameter in soybean cohorts than in sunflower cohorts suggests that body size is more constrained by plant size in soybean. Soybean also proved to be a substantially inferior adult diet for *D. texanus* compared to either wild or cultivated *H. annuus*, suggesting that beetles may exploit alternative food plants during the pre-reproductive period prior to invading soybean fields. Furthermore, cohorts from soybean with low body weights may experience a larval legacy of reduced longevity that is independent of adult diet. Although larval host plant did not affect female oviposition behavior, an adult diet of sunflower yielded females with higher levels of reproductive activity on sunflower than on soybean, and higher levels activity on sunflower than soybean-fed females, whereas females fed soybean had similar levels of reproductive activity on both host plants. Stable isotopes of nitrogen can be used to distinguish the larval host plant of adult beetles, regardless of adult diet, and this technique could be useful for estimating dispersal and recruitment rates of adults between soybean and sunflower when these crops are rotated in adjacent fields.
